# Innate Variability in Physiological and Omics Aspects of the Beta Thalassemia Trait-Specific Donor Variation Effects

**DOI:** 10.3389/fphys.2022.907444

**Published:** 2022-06-08

**Authors:** Alkmini T. Anastasiadi, Vassilis L. Tzounakas, Monika Dzieciatkowska, Vasiliki-Zoi Arvaniti, Effie G. Papageorgiou, Issidora S. Papassideri, Konstantinos Stamoulis, Angelo D’Alessandro, Anastasios G. Kriebardis, Marianna H. Antonelou

**Affiliations:** ^1^ Department of Biology, School of Science, National and Kapodistrian University of Athens (NKUA), Athens, Greece; ^2^ Department of Biochemistry and Molecular Genetics, School of Medicine, University of Colorado, Aurora, CO, United States; ^3^ Laboratory of Reliability and Quality Control in Laboratory Hematology (HemQcR), Department of Biomedical Sciences, School of Health and Welfare Sciences, University of West Attica (UniWA), Egaleo, Greece; ^4^ Hellenic National Blood Transfusion Centre, Athens, Greece

**Keywords:** genetic variability, storage lesion, beta-thalassemia trait, red blood cells, omics

## Abstract

The broad spectrum of beta-thalassemia (*β*Thal) mutations may result in mild reduction (*β* ^++^), severe reduction (*β* ^+^) or complete absence (*β* ^0^) of beta-globin synthesis. *β*Thal heterozygotes eligible for blood donation are “good storers” in terms of red blood cell (RBC) fragility, proteostasis and redox parameters of storage lesion. However, it has not been examined if heterogeneity in genetic backgrounds among *β*Thal-trait donors affects their RBC storability profile. For this purpose, a paired analysis of physiological and omics parameters was performed in freshly drawn blood and CPD/SAGM-stored RBCs donated by eligible volunteers of *β* ^++^ (N = 4), *β* ^+^ (N = 9) and *β* ^0^ (N = 2) mutation-based phenotypes. Compared to *β* ^+^, *β* ^++^ RBCs were characterized by significantly lower RDW and HbA_2_ but higher hematocrit, MCV and NADPH levels *in vivo*. Moreover, they had lower levels of reactive oxygen species and markers of oxidative stress, already from baseline. Interestingly, their lower myosin and arginase membrane levels were accompanied by increased cellular fragility and arginine values. Proteostasis markers (proteasomal activity and/or chaperoning-protein membrane-binding) seem to be also diminished in *β* ^++^ as opposed to the other two phenotypic groups. Overall, despite the low number of samples in the sub-cohorts, it seems that the second level of genetic variability among the group of βThal-trait donors is reflected not only in the physiological features of RBCs *in vivo*, but almost equally in their storability profiles. Mutations that only slightly affect the globin chain equilibrium direct RBCs towards phenotypes closer to the average control, at least in terms of fragility indices and proteostatic dynamics.

## Introduction

The storability profile of red blood cells (RBCs) seems to be highly dependent on intrinsic donor characteristics. Both genetic and environmental factors have been studied during the last decade in the context of donor variation effects upon storage and transfusion therapy. Donor’s sex (Szczesny-Malysiak et al., 2021), ethnicity (Kanias et al., 2017) and glucose-6-phosphate dehydrogenase (G6PD) activity (Tzounakas et al., 2016; Francis et al., 2020), as well as lifestyle aspects, such as smoking (Stefanoni et al., 2020) and caffeine or alcohol consumption (D'Alessandro et al., 2020a; D'Alessandro et al., 2020b) have been proven to affect differentially the storage and/or post-transfusion efficacy of donated RBCs. The same is true for RBCs with distinct hemoglobin (Hb) variants. For instance, stored RBCs from donors with elevated glycosylated Hb present increased susceptibility to lysis, phosphatidylserine (PS) externalization and non-reversible shape modifications (Li et al., 2022). Moreover, polymorphisms in *HbA2* gene are associated with a reduced hemoglobin increment (Roubinian et al., 2022), and sickle cell trait with increased storage hemolysis and removal in animal models of transfusion (Osei-Hwedieh et al., 2016).

RBCs from beta-thalassemia minor (*β*Thal^+^) eligible donors have been extensively studied lately with respect to their physiological, metabolic and proteomic profiles during storage. These cells seem to possess an intrinsic resistance to both spontaneous and induced lysis (Tzounakas et al., 2022), as well as an array of metabolic and proteomic features indicative of advantageous control of oxidative and proteotoxic stresses (Anastasiadi et al., 2021b; Tzounakas et al., 2021; Tzounakas et al., 2022). Regarding their post-transfusion aspects, stored βThal^+^ RBCs demonstrate resilience against lysis following exposure to plasma at body temperature, along with a trend for increased recovery post transfusion in mice recipients (Anastasiadi et al., 2021a). These superior post-storage phenotypes have been found linked to *β*Thal^+^-specific variations in baseline or storage parameters, such as cell fragilities, cytoskeleton composition, or urate (Anastasiadi et al., 2021a; Anastasiadi et al., 2022).

Besides divergence of specific donor cohorts from the average control, a range of within-group variation is also anticipated. In the case of beta-thalassemia the highly heterogenous genetic setting of mutations and related polymorphisms is translated to a broad spectrum of clinical and cellular phenotypes (Giardine et al., 2021) with variable prevalence within distinct national settings. More specifically, in Greece, mutations leading to severe reduction in *ß*-globin synthesis (*β* ^+^) represent almost 50% of the reported thalassemia alleles (Boussiou et al., 2008) followed by mutations resulting in null synthesis or slight reduction of *ß*-chains. Therefore, the cellular effects of each mutation might lead to a different storability phenotype. Having this in mind, the aim of the present study was to examine the innate variation of hematological, physiological, metabolic and protein parameters in freshly drawn and stored RBCs from a group of βThal^+^ donors stratified by the degree of *ß*-globin synthesis imposed by the affected allele.

## Materials and Methods

### Biological Samples and Blood Unit Preparation

Venous blood from fifteen regular βThal^+^ blood donors was collected into EDTA and citrate vacutainer tubes. The same subjects donated blood to prepare and store RBC units in citrate-phosphate-dextrose (CPD)/saline-adenine-glucose-mannitol (SAGM), for 42 days at 4°C. βThal^+^ trait was confirmed by Hb electrophoresis and molecular identification of mutations (IVS I-1, IVS I-6, IVS I-110, IVS II-1 and IVS II-745). The samples were subsequently categorized as *β* ^++^ (*n* = 4), *β* ^+^ (*n* = 9) and *β* ^0^ (*n* = 2) according to the impact that the mutations have upon the *ß*-globin synthesis (from slight -*β* ^++^- to severe -*β* ^0^- reduction in *ß*-globin levels). The RBC units were sampled every week under aseptic conditions. The study was approved by the Ethics Committee of the Department of Biology, School of Science, NKUA and investigations were carried out upon donor consent, in accordance with the principles of the Declaration of Helsinki.

### Hematological and Biochemical Measurements

BC-3000 PLUS, MINDRAY Celltac E, MEK-7222 Κ, NIHON KOHDEN automatic blood cell counters were used for complete blood count through double measurements to achieve maximum reliability, while the automatic analysers Hitachi 902, AVL Series Electrolyte Analyzer 9,180 and Elecsys Systems Analyzer (Roche Diagnostics, Risch-Rotkreuz, Switzerland) were used for the biochemical analysis of triglycerides, lipoproteins, iron (Fe), electrolytes and ferritin.

### Hemolysis Parameters

Storage hemolysis was calculated via spectrophotometry using Harboe’s method (Harboe, 1959) followed by Allen’s correction. For assessment of osmotic hemolysis, the samples were exposed to ascending concentrations of NaCl and then the mean corpuscular fragility (MCF) index (i.e., %NaCl at 50% hemolysis) was calculated. Mechanical hemolysis was estimated following rocking of RBCs with stainless steel beads for 1 h and measurement of the Hb released in the supernatant compared to non-rocked counterparts (Tzounakas et al., 2022).

### Reactive Oxygen Species Accumulation and Proteasome Activity

The intracellular accumulation of reactive oxygen species (ROS) was measured via fluorometry (BIORAD Hercules, CA, United States) by using the membrane permeable and redox-sensitive probe 5-(and-6)-chloromethyl-2′,7′-dichloro-dihydro-fluoresceindiacetate, acetyl ester (CM-H_2_DCFDA; Invitrogen, Molecular Probes, Eugene, OR, United States). This assay was performed with or without prior oxidative stimulation of RBCs by diamide (2 mM) or phenylhydrazine (PHZ; 100 μM) for 45 min at 37°C. Fluorometry was also used for the determination of caspase-like (CASP-like), chymotrypsin-like (CH-like) and trypsin-like (TR-like) proteasome activities in cytosol and membrane fractions. For this purpose, 120–200 μg of protein samples were incubated with the fluorogenic substrates Suc-Leu-Leu-Val-Tyr-aminomethylcoumarin (AMC) (CH-like), z-Leu-Leu-Glu-AMC (CASP-like), and Boc-Leu-Arg-Arg-AMC (TR-like) for 1.30 h (CH-like) or 3 h (CASP- and TR-like) at 37°C in the dark (Anastasiadi et al., 2021b). All substrates were produced from Enzo Life Sciences (New York, NY, United States). Fluorescent units were normalized to protein levels to reach a quantitative result.

### Metabolomics and Proteomics Analyses

For the metabolomics analysis, 100 μl of stored RBCs (or 20 μl of plasma/supernatants) were collected on a weekly basis, extracted at 1:6 (or 1:25) dilution in methanol:acetonitrile:water (5:3:2) and analyzed by UHPLC-MS (Ultimate 3000 RSLC-Q Exactive, Thermo Fisher), as previously described (D'Alessandro et al., 2019; Nemkov et al., 2019). Sample extracts (10 μl) were loaded onto a Kinetex XB-C18 column (150 mm × 2.1 mm × 1.7 μm—Phenomenex, Torrance, CA, United States). A 5-min gradient from 5 to 95% B (phase A: water +0.1% formic acid and B: acetonitrile +0.1% formic acid) eluted metabolites into a Q Exactive system (Thermo, Bremen, Germany), scanning in full MS mode or performing acquisition independent fragmentation (MS/MS analysis—5 min method) at 70,000 resolution in the 60–900 m/z range, 4 kV spray voltage, 15 sheath gas, and five auxiliary gas, operated in negative and then positive ion mode (separate runs). Metabolite assignment was performed against an in-house standard library, as reported (Nemkov et al., 2017), through the freely available software Maven (Princeton University, United States). No data pre-processing (neither normalization nor log-transformation) was performed. Proteomics analysis was performed on isolated membranes of early- and late-stored RBCs (*n* = 12 at each time point; *n* = 2 for *β* ^++^, *n* = 8 for *β* ^+^, *n* = 2 for *β* ^0^; obtained by hypotonic lysis). Samples (200 ng) were loaded onto individual Evotips (desalting) and were subsequently washed (20 μl 0.1% formic acid), followed by the addition of 0.1% formic acid to keep the Evotips wet. The Evosep One system was coupled to a timsTOF Pro mass spectrometer (Bruker Daltonics, Bremen, Germany). Data were collected over a m/z range of 100–1700 for MS and MS/MS on the timsTOF Pro instrument using an accumulation and ramp time of 100 ms. PEAKS studio (Version X+, Bioinformatics Solutions, Waterloo, ON, United States) was used for post-processing. The relative protein levels were normalized on the total amount of proteins.

### Statistical Analysis

All physiological experiments were performed in triplicate. Statistical analysis was performed by using the statistical package SPSS Version 22.0 (IBM Hellas, Athens, Greece, administered by NKUA). Between-group differences in freshly drawn blood (*in vivo* comparison) were assessed by independent *t*-test. Repeated measures ANOVA with Bonferroni-like adjustments for multiple comparisons was used for the evaluation of time-course and between groups differences in stored RBC units. Significance was accepted at *p* < 0.05. Due to the low number of samples analyzed by proteomics methods (distinguished by dashed boxes throughout the figures in order to highlight their mostly qualitative assessment) or those falling into the *β* ^0^ category (Figure 4) all selected parameters satisfied the criteria of both statistical significance (<0.05) and fold change (>1.25), with the exception of Piezo-1 that satisfies only the first criterion.

## Results

**FIGURE 1:**
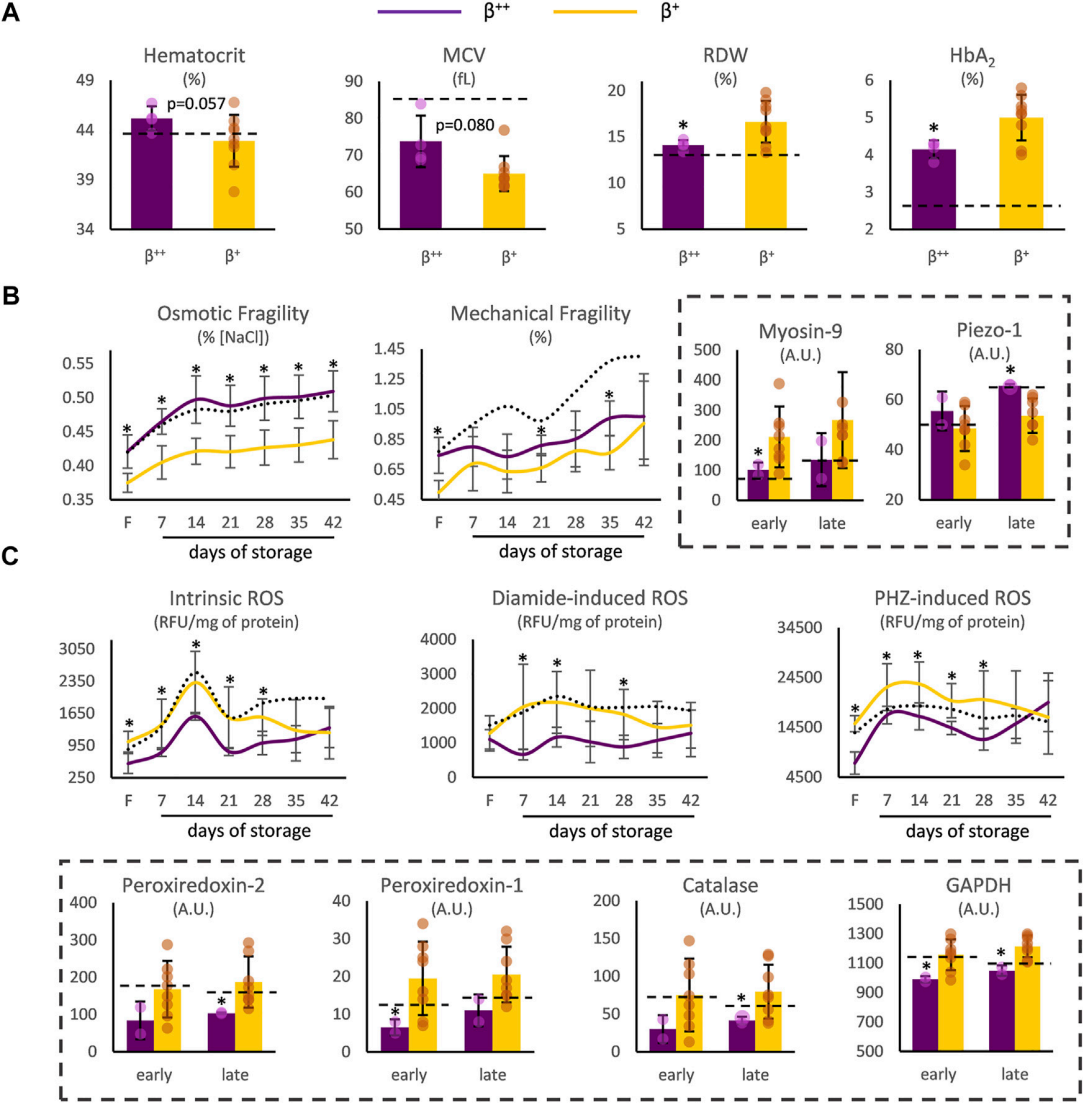
RBC indices and physiology differences between β^++^ and β^+^ heterozygotes. **(A)** Hematological indices in freshly drawn blood. **(B)** Cellular fragility and **(C)** redox parameters before and during storage. Values for average controls are shown by dashed lines. Proteomic parameters are shown in dashed boxes (*n* = 2 vs. 8, β^++^ vs. β^+^). (*) *p* < 0.05. F: freshly drawn blood; A.U, arbitrary units; ROS: reactive oxygen species; RFU: relative fluorescence units; PHZ: phenylhydrazine; GAPDH: glyceraldehyde-3-phosphate dehydrogenase.

At first, we focused on probable differences between *β* ^++^ (slight reduction in *ß*-globin) and *β* ^+^ (severe reduction but not null synthesis of *ß*-globin chains) samples. In freshly drawn blood, *β* ^++^ heterozygotes presented a trend toward higher hematocrit and mean corpuscular volume (MCV), but significantly lower red cell distribution width (RDW) and HbA_2_ (Figure 1A). Moving on to storage, while similar levels of spontaneous hemolysis were observed in *β* ^++^ and *β* ^+^ units (e.g., late storage: 7.42 ± 2.10 vs. 5.83 ± 2.81 mg Hb/dL, *β* ^++^ vs. *β* ^+^, *p* = 0.294), β^++^ RBCs presented increased osmotic fragility and sporadically increased mechanical fragility throughout storage, with both differences evident in freshly drawn blood as well (Figure 1B). At the same time, different levels of myosin-9 and piezo-1 proteins (that play a role in RBC deformability and volume regulation) were also detected in those groups (Figure 1B).

Concerning biochemical and proteomic features that vary as a function of the oxidative burden of RBCs, *β* ^++^ RBCs presented lower intracellular levels of ROS when compared to *β* ^+^, either intrinsic or induced by thiol- and hemoglobin-oxidizing agents, from the beginning until the middle of the storage period (Figure 1C). Interestingly, intrinsic, and phenylhydrazine-induced ROS already differed at baseline. The binding of redox-related proteins on the membrane was also distinct: members of the peroxiredoxin family, along with catalase and glyceraldehyde-3-phosphate dehydrogenase (GAPDH) were less evident in the isolated *β* ^++^ membranes (Figure 1C). It should be noted that no differences arose regarding membrane protein carbonylation (e.g., protein carbonylation index day 21: 36.73 ± 7.04 vs. 33.43 ± 9.12, *β* ^++^ vs. *β* ^+^, *p* > 0.05) and extracellular antioxidant capacity (e.g., total antioxidant capacity day 35: 428 ± 93 vs. 354 ± 64 μM Fe^2+^, *β* ^++^ vs. *β* ^+^, *p* > 0.05).

**FIGURE 2:**
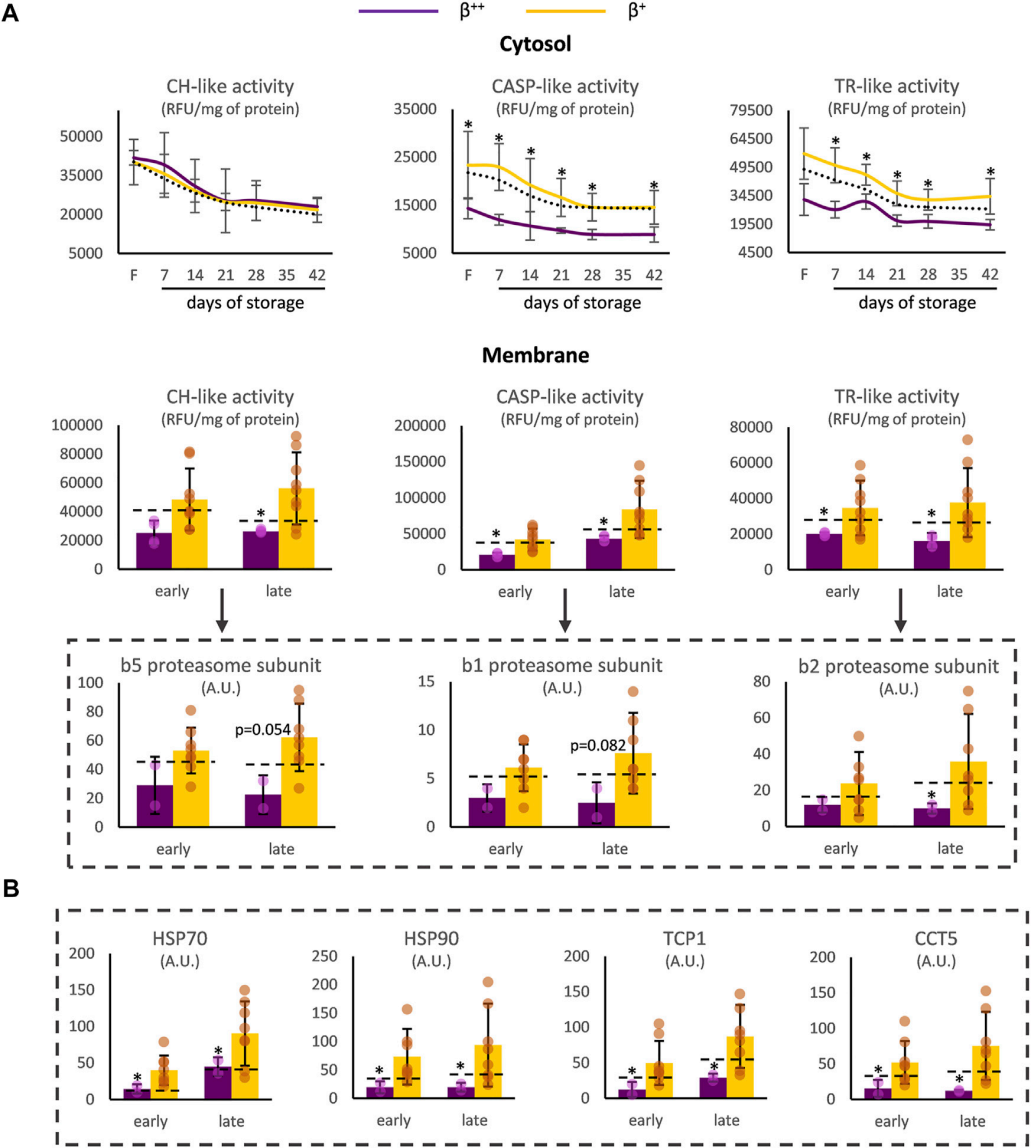
Proteostasis differences between *β* ^++^ and *β* ^+^ heterozygotes. **(A)** Proteasome activity in the cytosol and the membrane and binding of proteasome activity subunits on the membrane. **(B)** Binding of chaperoning proteins on the membrane. Values for average controls are shown by dashed lines. Proteomic parameters are shown in dashed boxes (*n* = 2 vs. 8, *β* ^++^ vs. *β* ^+^). (*) *p* < 0.05. F: freshly drawn blood; CH-like: chymotrypsin-like, CASP-like: caspase-like, TR-like: trypsin-like proteasome activities; RFU: relative fluorescence units; A.U, arbitrary units; HSP: heat shock protein; TCP1: T-complex protein one; CCT: T-complex subunit.

Proteostasis was also affected by the degree of *ß*-globin synthesis. In the cytosol of *β* ^++^, CASP- and TR-like (but not CH-like) activities were significantly reduced before (for TR-like activity at baseline *p* = 0.057) and during storage (Figure 2A) compared to the *β* ^+^ values. With regards to the membrane, all three proteasomal activities presented lower levels in *β* ^++^ vs. *β* ^+^ at both early and late storage. This finding was accompanied by a trend for lower binding of the b5, b1 and b2 proteasome subunits (where the three different proteolytic specificities of the proteasome are located) in the membrane, especially at late storage, in *β* ^++^ RBCs (Figure 2A). In parallel, heat shock proteins and components of the chaperoning T-complex also exhibited lower levels in the membranes of *β* ^++^ RBCs in comparison to *β* ^+^ (Figure 2B).

**FIGURE 3:**
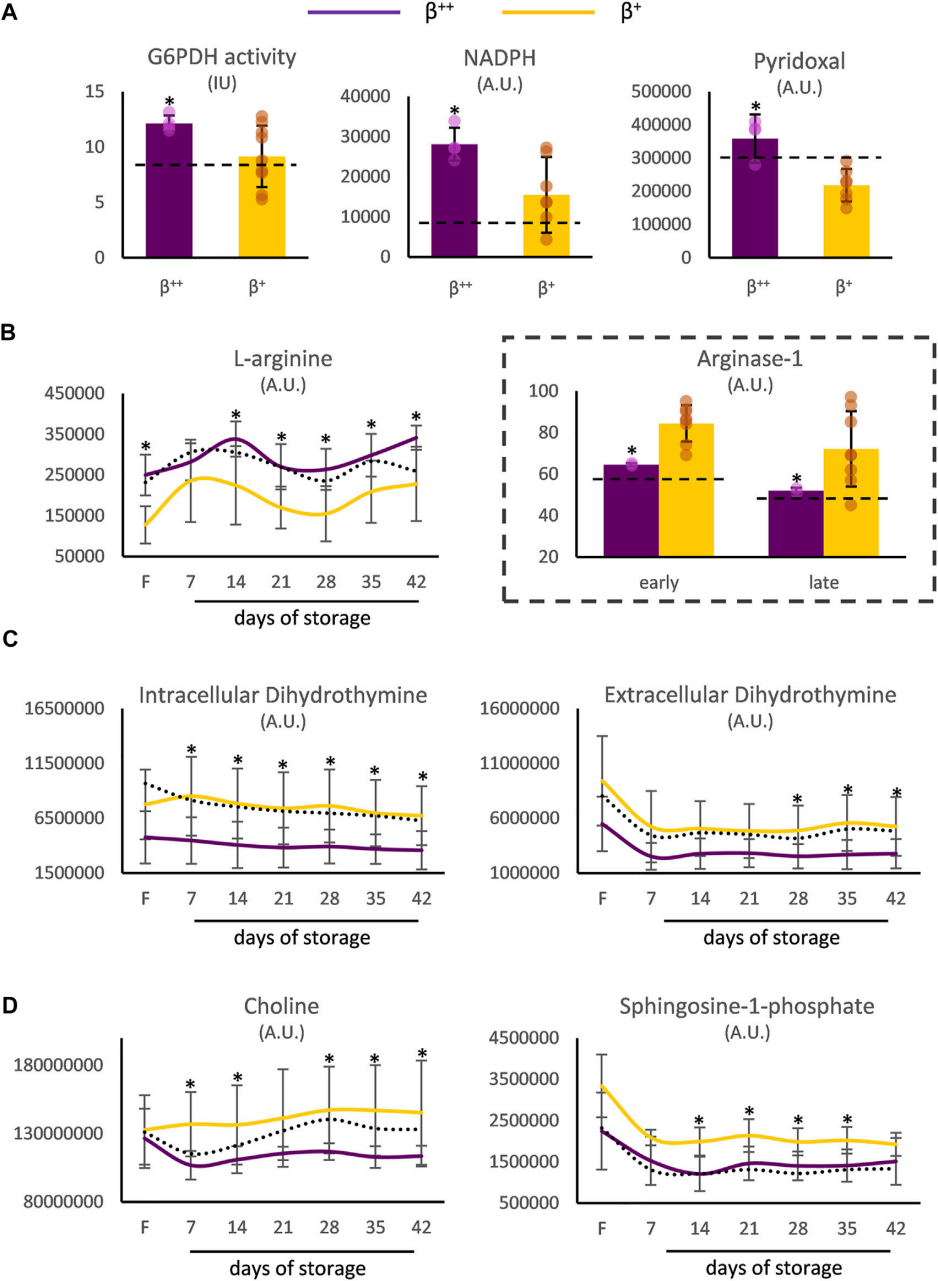
Metabolism differences between *β* ^++^ and *β* ^+^ heterozygotes. **(A)** *In vivo* differences in reducing and antioxidant powers. Differences in freshly drawn and stored red blood cells in **(B)** arginine, **(C)** dihydrothymine and **(D)** metabolites implicated in the biosynthesis of glycerophospholipids. Values for average controls are shown by dashed lines. Proteomic parameters are shown in dashed boxes (*n* = 2 vs. 8, *β* ^++^ vs. *β* ^+^). (*) *p* < 0.05. F: freshly drawn blood; G6PDH: glucose-6-phosphate dehydrogenase; IU: international units; NADPH: nicotinamide adenine dinucleotide phosphate; A.U. arbitrary units.

Moreover, the RBCs of *β* ^++^ donors demonstrated increased levels of G6PD activity and of the relevant metabolites NADPH and pyridoxal *in vivo* (Figure 3A). Both before and throughout storage, l-arginine stood out by presenting elevated values in *β* ^++^ vs. *β* ^+^ RBCs, probably in agreement with the lower membrane binding levels of arginase-1 (Figure 3B). Another metabolite that was differentially affected by the mutation’s severity was dihydrothymine. Either throughout storage (intracellularly) or in the last 2 weeks of it (extracellularly) lower levels of dihydrothymine were detected in the group of *β* ^++^ versus *β* ^+^ (Figure 3C). Finally, two metabolites implicated in the biosynthesis of glycerophospholipids, namely choline and sphingosine-1-phosphate (S1P), also showed lower levels in the same subgroup throughout the storage period (Figure 3D). All other metabolites tested presented similar levels between the two groups (e.g., urate day 7: 17 × 10^6^±6 × 10^6^ vs. 13 × 10^6^±5 × 10^6^ A.U., *β* ^++^ vs. *β* ^+^).

**FIGURE 4:**
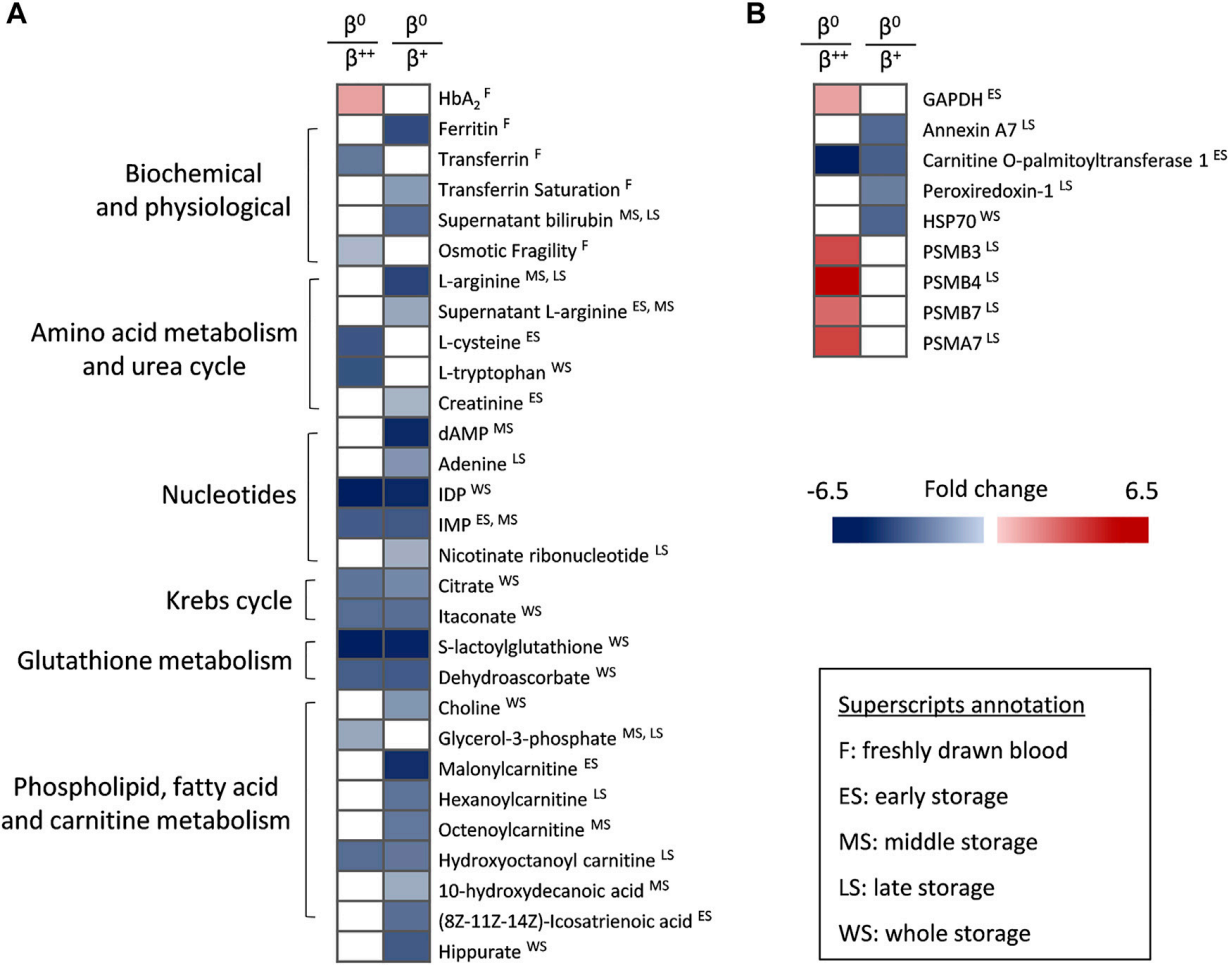
Statistically significant differences between *β* ^++^ or *β* ^+^ heterozygotes and *β* ^0^. **(A)** Biochemical, physiological, metabolic and **(B)** proteomic differences. All differences shown satisfy the criterion of 1.25 fold. n = 2 vs. 8 vs. 2, *β* ^++^ vs. *β* ^+^ vs. *β* ^0^. GAPDH: glyceraldehyde-3-phosphate dehydrogenase; HSP: heat shock protein; PSM: proteasome subunit.

Despite the low number of *β* ^0^ donors, that renders comparisons with the other groups mainly qualitative, the *β* ^0^ (null *ß*-globin synthesis) subjects were characterized by higher levels of HbA_2_ but lower levels of ferritin, transferrin and osmotic fragility compared to *β* ^++^ or *β* ^+^ donors *in vivo* (Figure 4A). In terms of metabolism, *β* ^0^ RBCs presented a downregulated amino acid metabolism sporadically during storage, as in the case of l-arginine (lower levels than in *β* ^+^) and l-tryptophan (lower levels than in *β* ^++^). Nucleotides such as IMP, carboxylic acids and glutathione-related metabolites were also decreased in *β* ^0^, mainly throughout storage and against both other donor groups. Additionally, molecules related to fatty acid and carnitine metabolism were found significantly lower in the same group, especially when compared to *β* ^+^ RBCs in specific storage periods (Figure 4A). Lastly, some proteins also exhibited distinct profiles in the *β* ^0^ RBC membrane (Figure 4B), including several proteasome subunits that along with GAPDH were upregulated during late or early storage, respectively, when compared to *β* ^++^. On the other hand, the presence of annexin A7 and peroxiredoxin-1 was minor in the membrane of late-stored *β* ^0^ in comparison to *β* ^+^. Another protein that differed among the three groups was carnitine O-palmitoyltransferase 1, the early levels of which gradually declined from the *β* ^++^ to the *β* ^0^ status (Figure 4B).

## Discussion

As previously shown, RBCs from *β*Thal^+^ donors can effectively cope with storage lesion, especially with regards to the storage-related hemolysis stress, maintaining at the same time enhanced proteostasis capacity and uric acid-related intracellular and extracellular antioxidant power. Hereby, we report a variation within the group of heterozygotes, with *β* ^++^ donors exhibiting worse RBC fragility indices but lower oxidative burden and proteasome activity than *β* ^+^. It was surprising to also find some *β* ^0^ subjects within the cohort of eligible donors, who showed unique nucleotide and amino acid metabolism features. A rather expected variation was found in hematological and biochemical data among the three sub-groups (*β* ^++^, *β* ^+^ and *β* ^0^) *in vivo*.

Silent mutations, which lie behind the *β* ^++^ phenotype, lead to just a small imbalance of the *α*-/*β*-globin synthesis ratio (Cao and Galanello, 2010), therefore, less excess of *α*-chains is present in cells. It is established that the accumulation of unpaired *α*-globin chains promotes the generation of ROS, thus, modifying the redox equilibrium of the cell and leading to oxidative damages (Olivieri, 1999). At the same time, RBCs in beta-thalassemia trait seem to possess an advantageous genetic regulation of antioxidant enzymes, leading to upregulated expression of peroxiredoxin-2 and superoxide dismutase (Teran et al., 2020). Our cohort of beta-thalassemia carriers, in contrast to previously studied groups (Selek et al., 2007), also exhibits higher than average extracellular antioxidant capacity, as well as superior intracellular equilibrium of redox metabolites (e.g., increased urate, decreased s-allantoin) (Tzounakas et al., 2022), irrespectively to the variable degree of beta-globin synthesis imposed by the heterozygous state of β^++^ and β^+^ underlying mutations. It is tempting to hypothesize that the slight excess of α-globin chains in *β* ^++^ vs. *β* ^+^ subjects, along with the boosted antioxidant system observed in both subgroups, altogether provide the first with an advantage regarding the control of ROS accumulation. The above hypothesis is also supported by the susceptibility to PHZ- (and thus, to oxidized-Hb) induced ROS production: while overall similar to the control (when all mutations analyzed as a group), when stratified in the currently examined subgroups *β* ^+^ RBCs present sporadically higher but *β* ^++^ lower susceptibility to ROS elevation (e.g., day 28: 20,079 ± 5,267 vs. 16,165 ± 4,529 vs. 12,058 ± 1,946 RFU/mg of protein, *β* ^+^ vs. control vs. *β* ^++^, *p* < 0.05). Moreover, while the storage levels of ROS in the *β* ^+^ subgroup followed the general variation pattern observed in heterozygous versus control samples, namely lower levels at late-storage (Tzounakas et al., 2022), the levels of *β* ^++^ RBCs were inferior throughout the storage period (e.g., day 14: 2,321 ± 678 vs. 2,539 ± 789 vs. 1,584 ± 83 RFU/mg of protein, *β* ^+^ vs. control vs. *β* ^++^, *p* > 0.05 only for *β* ^+^ vs. control). In this context, it would be very interesting to study the redox equilibrium of RBCs from the minority group of *β* ^0^ eligible donors during the storage period.

Following binding to the RBC cytoskeletal network, the free α-globin chains tend to autoxidize and cause massive membrane oxidative damage (Ficarra et al., 2009). The enhanced proteostatic system of *β* ^+^ and *β* ^0^ cells, observed through the elevated levels of proteasomal activity and/or the increased binding of chaperone and proteasome proteins to the membrane, seems capable to ameliorate the detrimental effects of oxidative stress upon the membrane. Indeed, we found no difference between the levels of membrane protein carbonylation between the distinct sub-groups. The proteasome machinery is abundant in the beta-thalassemic precursor cells to decongest the cell from the free α-globin chains (Khandros et al., 2012; Rivella, 2012), while in mature RBCs the same supramolecular complex appears to be involved in the degradation of oxidized Hb (Abi Habib et al., 2020). Regarding the latter, it has been indicated that several chaperones are also involved in α-globin detoxification (Khandros et al., 2012). The simultaneous recruitment of antioxidant cytosolic proteins such as peroxiredoxin-2 (Cho et al., 2014) in the membrane of *β* ^+^ RBCs (which is considered a response to local oxidative stress) (Rocha et al., 2009), might also assist in the overall membrane protection, especially considering the remarkable crosstalk between the redox and proteostasis networks in βThal^+^ stored RBCs (Anastasiadi et al., 2021b). It should not be omitted that peroxiredoxin-2 competes with hemichromes for Band 3 binding and can prevent the latter’s clustering and formation of senescence neo-antigen (Bayer et al., 2016). Nonetheless, excess of hemichromes, as shown in RBCs of βThal mice models can displace peroxiredoxin-2 from the membrane, highlighting the excessive oxidative challenges (Matte et al., 2010). Thus, there might be a fine line regarding the recruitment of peroxiredoxin-2 to the membrane under different levels of oxidative stress. In our case, the increased oxidative stress of β^+^ RBCs, that is additionally burdened during the stressful storage period, seems not to be translated to extreme membrane damage and subsequent cell lysis, since the βThal^+^ sub-groups presented similar spontaneous hemolysis levels. In striking contrast, in beta-thalassemia intermedia and major RBCs, the severe defects of the membrane components, caused by the precipitation of the unstable α-globin chains, contribute to hemolysis (Romanello et al., 2018). The increased binding of the cytosolic GAPDH to the membranes of β^0^ and β^+^ RBCs further highlights the oxidative stress imposed by the unmatched α-globin chains, since the relocation of GAPDH is observed under pro-oxidant conditions during storage and triggers glucose consumption through the pentose phosphate pathway to supply RBCs with reducing power (Reisz et al., 2016).

It has been previously shown that the α-globin related oxidation of membrane and skeletal proteins in thalassemic RBCs affects their mechanical stability (Schrier and Mohandas, 1992). The augmented proteostasis at the membrane level along with the observed variation in the membrane association of myosin proteoforms and piezo-1 protein could take the credit for the superior fragility of β^+^ versus β^++^ RBCs. Indeed, non-muscle myosin IIA plays a significant role in the control of RBC shape and deformability (Smith et al., 2018), while piezo-1 is involved in the regulation of cell volume (Svetina et al., 2019). Moreover, it has been shown that the membrane localization of peroxiredoxin-2 promotes K^+^ efflux through activation of the Gardos channel (Low et al., 2008), benefitting the cell in terms of osmotic stress tolerance. To further support this, elevated RBC osmotic fragility has been previously shown in Gardos-knockout mice (Grgic et al., 2009).

Only a few differences were detected in the levels of RBC metabolites between the three βThal^+^ subgroups, revealing that the metabolism of RBCs is rather compact in the thalassemia trait. Freshly drawn RBCs from *β* ^++^ subjects presented a redox equilibrium advantage compared to the *β* ^+^ counterparts, since they were enriched in NADPH −the driving force of several antioxidant pathways in RBCs− and the redox-related pyridoxal (Jain and Lim, 2001), a finding that is in line with the lower levels of ROS accumulation observed in this subgroup. Arginine was found in gradually lower levels from the *β* ^++^ towards the *β* ^0^ stored RBCs. It is known that arginine metabolism is dysregulated in thalassemia (Jain and Lim, 2001), while increased arginase-1 expression (currently observed in *β* ^+^ versus *β* ^++^) and activity, in parallel with low l-arginine levels have been linked to oxidative stress and hemolysis (Morris et al., 2017; Contreras-Zentella et al., 2019). In this context, the arginase-1 enriched *β* ^+^ RBCs also presented increased intrinsic oxidative burden when compared to *β* ^++^. During aging-related oxidative stress, arginase-1 is elevated (at least in animal models (Pandya et al., 2019)), thus the successively higher α-globin chain accumulation in the βThal^+^ subgroups might play a role in the differential arginine metabolism. Another metabolite that distinguished *β* ^++^ from *β* ^+^ was dihydrothymine. Interestingly, this pyrimidine metabolite that has been associated with the beneficial osmotic stability of βThal^+^ RBCs in previous studies (Anastasiadi et al., 2022) is currently found increased in the least fragile group. Nonetheless, the mechanistic basis (if any) underlying these observations needs further examination. Of note, the elevated levels of spingosine-1-phosphate in *β* ^+^ compared to *β* ^++^ stored RBCs, a lysophospholipid related to RBC energy metabolism regulation (Sun et al., 2016) and transfusion biology (Selim et al., 2011), predispose for favorable storability profile under hypoxic storage conditions. To support this, a previous study on G6PD deficient donors demonstrated significant correlations between baseline spingosine-1-phosphate levels and quality characteristics of stored RBCs (Reisz et al., 2017). Finally, *β* ^0^ RBCs presented decreased metabolites of the carboxylic acid, nucleotide, fatty acid and glutathione pathways when compared to the other two subgroups. RBCs from beta-thalassemic patients are characterized by decreased glutathione levels (Kalpravidh et al., 2013) and, therefore, sustained oxidative stress that can be ameliorated by inhibitors of glutathione efflux transporters (Muanprasat et al., 2013). In the same context, serum from beta-thalassemic subjects demonstrates down-regulated fatty acid metabolism (Musharraf et al., 2017), whereas carboxylic acids and metabolites of the arginine and glutathione routes were found slightly or significantly downregulated in the lungs of murine models of beta-thalassemia (Buehler et al., 2021). According to these findings, it appears that the metabolism features of *β* ^0^ RBCs are closer to the thalassemia disease profile, even though the currently studied *β* ^0^ heterozygotes were considered eligible blood donors. Again, the metabolic profile of donated RBCs from the *β* ^0^ subgroup deserves further examination by studies in bigger cohorts.

Overall, while presenting some solid characteristics that differentiate them from control RBCs, including the highly important superior end-of-storage hemolysis and antioxidant arsenal (such as urate), βThal^+^ stored RBCs also exhibit an inside-group variation. It is plausible to suspect that this variation is *α*-globin excess-dependent, since milder mutations lead to phenotypes closer to the average control, while severe mutations tend to phenotypic features that are closer (at some level) to the disease state. One limitation of this study that does not allow drawing of broad hypotheses is the low number of donors enrolled, especially in the group of *β* ^0^ subjects, and the small number of samples used for proteomics analyses. However, this is the first time that the special features of the βThal^+^ donor subgroups are reported in the research field of RBC transfusion and donor variation effects. In fact, it would be really interesting and helpful to design large-scale studies, with βThal^+^ subgroups exhibiting wider genetic heterogeneity. Such targeted research could give us the ability to draw more sound conclusions regarding the relation between the magnitude of the thalassemia imprint on RBCs and their storage quality metrics.

## Data Availability

The raw data supporting the conclusion of this article will be made available by the authors, without undue reservation.
